# Improving medical certification of cause of death: effective strategies and approaches based on experiences from the Data for Health Initiative

**DOI:** 10.1186/s12916-020-01519-8

**Published:** 2020-03-09

**Authors:** John D. Hart, Renee Sorchik, Khin Sandar Bo, Hafizur R. Chowdhury, Saman Gamage, Rohina Joshi, Viola Kwa, Hang Li, Buddhika P. K. Mahesh, Deirdre Mclaughlin, Lene Mikkelsen, Janet Miki, Roderick Napulan, Rasika Rampatige, Matthew Reeve, Carmina Sarmiento, Nang Su War, Nicola Richards, Ian D. Riley, Alan D. Lopez

**Affiliations:** 1 Melbourne School of Population and Global Health, The University of Melbourne, Carlton, Victoria 3053 Australia; 2 The George Institute for Global Health, UNSW Sydney, Newtown, New South Wales 2042 Australia; 3 Civil Registration and Vital Statistics, Vital Strategies, Bloomberg Data for Health Initiative, Santiago de Surco, Lima Peru; 4 Health Facility Development Bureau, Department of Health, Manila, Philippines; 5 John Snow, Inc. (JSI Research & Training Institute, Inc.), Mandalay, Myanmar

**Keywords:** Cause of death, Certification, Certificate, Mortality, Training

## Abstract

**Background:**

Accurate and timely cause of death (COD) data are essential for informed public health policymaking. Medical certification of COD generally provides the majority of COD data in a population and is an essential component of civil registration and vital statistics (CRVS) systems. Accurate completion of the medical certificate of cause of death (MCCOD) should be a relatively straightforward procedure for physicians, but mistakes are common. Here, we present three training strategies implemented in five countries supported by the Bloomberg Philanthropies Data for Health (D4H) Initiative at the University of Melbourne (UoM) and evaluate the impact on the quality of certification.

**Methods:**

The three training strategies evaluated were (1) training of trainers (TOT) in the Philippines, Myanmar, and Sri Lanka; (2) direct training of physicians by the UoM D4H in Papua New Guinea (PNG); and (3) the implementation of an online and basic training strategy in Peru. The evaluation involved an assessment of MCCODs before and after training using an assessment tool developed by the University of Melbourne.

**Results:**

The TOT strategy led to reductions in incorrectly completed certificates of between 28% in Sri Lanka and 40% in the Philippines. Following direct training of physicians in PNG, the reduction in incorrectly completed certificates was 30%. In Peru, the reduction in incorrect certificates was 30% after implementation and training on an online system only and 43% after training on both the online system and basic medical certification principles.

**Conclusions:**

The results of this study indicate that a variety of training strategies can produce benefits in the quality of certification, but further improvements are possible. The experiences of D4H suggest several aspects of the strategies that should be further developed to improve outcomes, particularly key stakeholder engagement from early in the intervention and local committees to oversee activities and support an improved culture in hospitals to support better diagnostic skills and practices.

## Background

Accurate and timely data on cause of death (COD) is perhaps the most critical information source for guiding health programmes and policies [1]. COD data is essential for measuring how most health conditions are changing, both with respect to magnitude and distribution in a population [2]. Information on the national pattern of mortality is critically important for national and international health policy and planning, and forms a key measure of development progress. Further, monitoring of country progress towards the Sustainable Development Goals (SDGs) will be impossible without reliable mortality and COD data provided by civil registration and vital statistics (CRVS) systems: 7 goals and 17 of their corresponding indicators require cause-specific mortality data, the optimal source of which are functioning CRVS systems [3].

Population-based, cause-specific mortality statistics are generated from information provided on individual medical certificates of cause of death (MCCOD). The ‘gold standard’ for COD reporting is for a physician to certify the underlying COD based on the rules and procedures outlined in the International Statistical Classification of Diseases and Related Health Problems, currently in its 10th revision (ICD-10) [4]. Routine and correct application of the World Health Organization (WHO)-recommended international MCCOD form, as well as coding the data to ICD-10 standards, is essential for the production of reliable mortality statistics that are comparable between countries, for national and subnational populations, and for specific population groups over time [5].

There are three critical requirements on the information pathway from the notification or registration of a vital event, to generation of a vital statistic [6], namely (1) the ability of physicians to accurately complete a MCCOD, (2) the availability of trained mortality coders to accurately code information provided on MCCODs and identify the underlying COD, and (3) effective data consolidation procedures and statistical standards that transform individual codes into national mortality statistics. This paper focusses on experience from a multi-country initiative to improve the first of these components through training physicians to accurately complete the MCCOD.

The accuracy of medical certification of COD depends on many factors, including the certifiers’ knowledge and skills in correctly identifying the sequence of events leading to death and their understanding of the concept of the underlying COD [7]. The public health importance of accurate COD information, the concept of the underlying COD, the sequence of events leading to death, and how to correctly complete the MCCOD are rarely introduced to medical students [8]. Previous research, as well as experiences under the Bloomberg Data for Health (D4H) Initiative, indicates that the training on certification of COD provided to medical school students is inadequate globally [5]. As well as insufficient time dedicated to the topic, the medical curriculum in both high income and low- to middle-income countries (LMICs) is often taught from the viewpoint of legal or forensic medicine, rather than emphasising the public health importance of correct COD certification. This almost certainly has an impact on the information that physicians collect on MCCODs and ultimately on their diagnostic accuracy.

Given this situation, it is perhaps not surprising that the quality of national mortality statistics, where they have been formally evaluated, is often found to be low [9–12]. As many as half of all registered deaths globally do not have an accurate COD assigned [13]; and whilst the majority of LMICs do not have complete and reliable COD data [14], issues around data quality are global [15, 16]. A 2017 study at a selection of hospitals with high inpatient death rates in the USA found that 46% of reviewed MCCODs were completed incorrectly [17]. Similarly, in 2010, about 24% of deaths in South Africa were reported as being due to ill-defined or unknown causes, resulting in suboptimal and biassed COD information for planning purposes [12].

Many countries are beginning to implement training for physicians on the correct completion of MCCOD, as well as introducing regular monitoring of MCCOD quality at hospitals [18, 19]. However, there is very little evidence available on the best training strategies to improve medical certification and ensure sustainability. A key priority of D4H has been to improve the efficiency, comparability, and quality of COD information routinely reported on MCCODs by physicians. This included support for establishing national mortality committees; introducing the international standard MCCOD form into hospitals; developing resources and tools, such as digital applications, training videos, and manuals; providing training courses on certification and supporting the integration of training into medical curricula; and periodically assessing the quality of certification and impact of training strategies. This paper reports on the different country implementation strategies implemented under the D4H Initiative to provide training and education on MCCOD, including an evaluation of effectiveness.

## Methods

Under D4H, a range of intervention strategies for MCCOD training, education, and advocacy were employed. We describe below the three strategies rolled out in five countries (Myanmar, Papua New Guinea, the Philippines, Peru, and Sri Lanka) as examples of the range of options available to improve medical certification practices, and how they also need to be tailored to country context and circumstances: (1) training of trainers (TOT), (2) direct training, and (3) online and basic training. In addition to the specific strategies in countries, technical expertise, information, products, and technological advancements to support improvements in completing MCCODs were provided to countries by the University of Melbourne (UoM) D4H and made available on the CRVS Knowledge Gateway [20].

### Training of trainers strategy

The main strategy involved a TOT approach, whereby UoM D4H staff trained key trainers in the country, who then provided further trainings to colleagues in (primarily) government hospitals. Three of the five country strategies described here used variations of the TOT approach, namely the Philippines, Myanmar, and Sri Lanka. The second strategy in Papua New Guinea (PNG) involved direct training of physicians in the main hospitals by UoM D4H staff. Finally, in Peru, a different strategy was employed, combining training on the use of an online system for medical certification with face-to-face training on completion of MCCODs. Variations of these strategies and country contexts are described below.

In the Philippines, a 2-day intensive training with 4 to 5 physicians per government hospital was held on correct ICD-compliant medical certification to produce a group of master trainers who would be able to conduct MCCOD training and regularly monitor MCCOD quality. An additional topic on coaching was included in one batch to improve the trainees’ capability to effectively deliver the training to other physicians. After the master training programme, trained hospital medical record staff were expected to assess a sample of 100 pre-training MCCODs from their hospital as well as post-training certificates, to assess improvement in the quality of completion of MCCOD. Two master training workshops were held, including training of physicians on medical certification and training of medical records staff on assessment of the quality of certification. Medical records staff were also provided with training on medical terminology to enhance their capability to review the death certificates. A system for regular assessment of death certificates was also piloted in some trained hospitals to ensure quality assurance sustainability in larger capacity hospitals.

In Myanmar, the intervention involved first establishing a technical working group to oversee MCCOD activities. In the early phase, 122 master trainers were trained from 58 hospitals across 1 state, 2 regions, and Nay Pyi Taw Council (Sagaing, Magway and Mon, and Nay Pyi Taw). In the next phase, 114 master trainers were trained from a further 56 hospitals across 11 regions, to conduct multiplier trainings in 56 hospitals across all 6 states and 5 regions. Advocacy meetings with hospital staff were held in 9 hospitals, and multiplier trainings were conducted in the regions, achieving a coverage of 80% of physicians. MCCOD training was introduced into the medical curriculum in 5 medical universities and 2 public health universities in Myanmar.

In Sri Lanka, five large hospitals were selected on the basis that they were already using the newly revised international standard MCCOD form. In late 2016, five master trainers were trained from each hospital and were required to roll out the training within the hospital over a 6-month period, from November 2016 to April 2017. Post-assessments were done in July 2017. Master trainers were given the necessary training to regularly assess samples of pre- and post-training MCCODs from their respective hospitals.

### Direct training strategy

Prior to the D4H intervention, very few deaths were certified in PNG. As such, UoM D4H’s objective in PNG was to facilitate the development of a comprehensive mortality surveillance system, including MCCOD information and COD for home deaths using verbal autopsy, partnering with the Department of Health in provinces chosen to trial the implementation of a new electronic National Health Information System (eNHIS). Improving medical certification initially involved ad hoc training of physicians on an opportunistic basis, for example, at medical symposia gatherings, but later became more focussed on direct training of physicians at six of the largest hospitals across the country. All trainings were provided by UoM D4H staff, with refresher trainings and monitoring of quality post-training supported locally at the hospitals through medical supervisors and hospital committees. Advocacy meetings were held regularly with senior physicians at each site and at the Department of Health as part of this strategy, and later in the intervention, MCCOD training was introduced into the medical curriculum at the main medical school.

### Online system and basic training strategy

The final country strategy employed to improve quality of medical certification was implemented in Peru, where two specific interventions were introduced to improve the completeness of death registration and the quality of COD data [19]. This involved firstly, in August 2016, the introduction of an online death notification and certification system, Sistema Informático Nacional de Defunciones (SINADEF), into health facilities and morgues. Secondly, the Ministry of Health introduced a training programme for physicians that included how to complete the MCCOD. A core team of trained physicians and other professionals trained physicians and statisticians at the main hospitals and morgues in how to use SINADEF. Trainings included 1 h on the online system and 1 h on the completion of the MCCOD. The total amount of time available was limited to 2 h because all health facilities considered this as the maximum amount of time that they could release doctors from clinical duties. Ideally, the MCCOD training alone would take a half to full day.

### Evaluation and assessment tool

Evaluation of the impact of each strategy was assessed by analysing MCCODs using a standardised rapid assessment tool [21]. A baseline assessment of quality was conducted on MCCODs collected from hospitals prior to the training, and a post-training assessment was conducted on MCCODs approximately 6 months to 2 years after training, aiming to assess knowledge retention over this time. For the evaluation in Peru, quality of completion of MCCODs was assessed for (1) physicians with no training on medical certification and who continued to complete paper forms and (2) physicians with training on either how to use the online system only (online intervention) or how to use the online system and basic medical certification principles (online and training intervention). The assessment tool was developed for use in D4H countries and designed to quickly assess the quality of medical certification of COD practices by checking for the presence of common errors in MCCODs. In addition to serving as the basis for the evaluation of impact, the tool can also be used to assess the quality of medical certification as part of routine assessment or to assess the training needs of physicians in designing COD certification training [21, 22].

The assessment tool is essentially a checklist of the most common errors that are seen in MCCODs. The assessment criteria are categorised as ‘major’ and ‘minor’, as shown in Table 1, depending on the likely impact that the error may have on the final selection of the underlying COD by the mortality coder. If the risk of misidentification by coders is deemed to be high, the error is classified as major. For example, recording multiple causes per line is classified as a major error as this error is likely to make it difficult for coders to apply selection and modification rules for selecting the underlying COD. Conversely, the presence of blank lines is likely to have a minor impact on the process of underlying COD selection and so is classified as a minor error.

**Table 1:** Classification of major and minor errors on medical certificates of cause of death

Error type	Description and implications
Major errors	
Multiple causes per line	The WHO ICD guidelines state that only one cause should be recorded per line in a medical certificate of cause of death (MCCOD). When more than one cause is reported on a single line, there will be more than one potential sequence, making it difficult for coders to identify the correct sequence of events leading to death, thus making it more difficult to select the correct underlying cause of death (UCOD).
Incorrect sequence of events leading to death	Mortality statistics are based on the UCOD, which is the condition or injury that initiated the sequence of events that led directly to death. When a clinically improbable sequence of events is recorded, it is impossible to select the correct UCOD.
Illegible handwriting	Illegible handwriting makes it difficult to determine if the sequence of events leading to death is probable or if an ill-defined condition is entered as an underlying cause. Illegible handwriting can also prevent coders from selecting the UCOD, rendering the MCCOD unusable for statistical purposes.
Ill-defined or poorly specified condition entered as the underlying COD	Ill-defined or poorly specified conditions are of no value for public health priority setting and do not provide any information for decision-makers about the comparative need for specific diseases and injury prevention programmes. These include, for example, organ failure (e.g. hepatic or cardiac failure), symptoms or signs (e.g. hematemesis, dyspnoea, fever), mode of dying (e.g. cardiac arrest, respiratory arrest), pathophysiological findings (shock), and others (trivial diseases such as colds, rhinitis).
Insufficient information on the external COD	Sufficient detail should be provided to identify the UCOD (e.g. circumstances, intent or nature of the accident or violence).
Insufficient information on neoplasms	Sufficient detail should be provided regarding the neoplasm (e.g. site, whether benign or malignant).
Minor errors	
Presence of blank spaces within the sequence of events	In completing the MCCOD, the certifier should use consecutive lines in part 1 of the certificate starting at line 1a. The UCOD should be recorded in the lowest used line of part 1. There should not be any blank lines within the sequence/chain of events leading to death.
Abbreviations used in certifying the death	Doctors are encouraged not to use abbreviations when certifying deaths as abbreviations can mean different things to different people. There is a chance that coders may misinterpret the abbreviation and code the death to a non-relevant cause.
Absence of disease time interval	The time interval should be entered for all conditions reported on the MCCOD, especially for the conditions reported in part 1. Time intervals are very important for correctly coding certain diseases and provide a check on the accuracy of the reported sequence of conditions.
Additional errors on the certificate	There may be other additional errors on MCCODs failing to identify pregnancy and maternal deaths.

## Results

### Training of trainers strategy

In all countries that implemented the main TOT intervention strategy, there were improvements in the quality of medical certification from 6 months to 2 years following training, detailed in Table 2. In the Philippines, a total of 1934 MCCODs (975 at baseline and 959 post-training) collected between 2016 and 2019 from 10 hospitals were used in the assessment. Prior to the training, 73% of MCCODs contained at least 1 major or minor error, decreasing to 44% post-training (Table 2). The presence of any major errors decreased by 46% post-training.

**Table 2:** Quality assessment of medical certificates of cause of death pre- and post-training in the Philippines, Myanmar, and Sri Lanka (training of trainers strategy)

Assessment	Philippines			Myanmar			Sri Lanka		
	MCCODs with errors (%)			MCCODs with errors (%)			MCCODs with errors (%)		
	Pre-training (*N* = 975)	Post-training (*N* = 959)	Percentage improvement*	Pre-training (*N* = 595)	Post-training (*N* = 600)	Percentage improvement*	Pre-training (*N* = 517)	Post-training (*N* = 558)	Percentage improvement*
Incorrectly completed certificates (at least one error)	**72.9**	**43.6**	**40.1**	**99.8**	**74.8**	**33.4**	**95.4**	**68.5**	**28.2**
Certificates with one error	37.2	28.4	23.6	21.7	23.7	−9.2	25.9	32.3	−24.7
Certificates with two or more errors	35.7	15.3	57.1	78.2	51.2	34.5	69.4	36.2	47.8
Certificates with at least one major error^†^	**41.6**	**22.6**	**45.6**	**63.2**	**44.8**	**29.1**	**58.8**	**37.5**	**36.2**
Certificates with minor errors only	**31.3**	**21.1**	**32.5**	**36.6**	**30.0**	**18.0**	**36.6**	**31.0**	**15.3**
Certificates with major errors									
Multiples causes per line	21.2	6.0	71.6	24.4	10.8	55.7	38.9	20.8	46.5
Incorrect sequence of events leading to death	27.1	12.4	54.2	7.9	5.8	26.5	37.1	17.0	54.1
Illegible handwriting	0.8	1.1	− 37.5	4.2	2.8	33.3	0.6	0.0	
Ill-defined condition entered as an underlying cause of death	28.6	15.5	45.8	44.5	32.7	26.5	4.4	10.6	140.9
Additional information on external cause not available	4.8	1.2	75.0	–	–	–	–	–	–
Additional information on neoplasms not available	2.3	1.9	17.0	1.4	0.3	78.5	4.3	0.5	95.0
Certificates with minor errors									
Presence of blank lines within the sequence of events	–	–	–	0.2	0.3	50.0	2.1	2.7	− 28.6
Abbreviations used in certifying the death	7.1	0.8	88.7	50.8	31.0	38.9	36.0	20.3	43.6
Absence of disease time interval	37.4	23.7	36.6	93.4	65.3	30.1	87.0	53.2	38.8
Additional errors on the certificate	5.3	1.1	79.2	1.6	0.7	56.2	2.9	0.2	93.1

Total column percentages may total > 100% as a single MCCOD may contain more than one error

^–^No data available

*Negative values indicate lower quality after training

^†^Category may contain minor errors

In Myanmar, a total of 1195 MCCODs (595 at baseline and 700 post-training) from 6 hospitals were reviewed between 2017 and 2018. Prior to training, 100% of MCCODs were completed incorrectly, decreasing to 75% after training. Excluding the disease time interval was the most common error prior to training, occurring in 93% of MCCODs, with incorrect sequencing as the least common error, occurring in just 8% of certificates. The presence of all major errors decreased by between 27 and 79% after training.

In Sri Lanka, a total of 1075 MCCODs were examined (517 at baseline and 558 post-training) during 2016 and 2017. At baseline, 95% of certificates were incorrectly completed and 59% contained major errors; this improved to 69% and 38%, respectively, post-training. The presence of any major errors decreased by 36% post-training.

### Direct training strategy

For the evaluation of the strategy employed in PNG, where country physicians were trained by UoM D4H staff, a total of 1326 MCCODs (948 at baseline and 378 post-training) collected between 2017 and 2019, from 3 hospitals, were used in the assessment. Post-training assessment took place in February 2019 using MCCODs completed approximately 6 months to 2 years post-training. Prior to the training, 86% of certificates were incorrectly completed, decreasing to 61% post-training (Table 3). The presence of major errors decreased by 45% after physicians received the training. There were reductions in each of the major errors assessed between 49 and 63%. Improvements were also seen among minor error categories, including a 73% reduction in the use of abbreviations.

**Table 3:** Quality assessment of medical certificates of cause of death pre- and post-training in PNG (direct training strategy)

Assessment	MCCODs with errors (%)		
	Pre-training (*N* = 948)	Post-training (*N* = 378)	Percentage improvement*
Incorrectly completed certificates (at least one error)	**86.4**	**60.6**	**29.8**
Certificates with one error	25.3	35.3	− 39.5
Certificates with two or more errors	61.1	25.3	58.59
Certificates with at least one major error^†^	**55.6**	**30.7**	**44.7**
Certificates with minor errors only	**30.8**	**29.9**	**2.92**
Certificates with major errors			
Multiples causes per line	16.3	7.9	51.5
Incorrect sequence of events leading to death	41.7	20.3	51.3
Illegible handwriting	4.3	1.6	62.8
Ill-defined condition entered as an underlying cause of death	39.1	18.7	52.1
Additional information on external causes not available	–	–	–
Additional information on neoplasms not available	4.5	2.3	48.8
Certificates with minor errors			
Presence of blank lines within the sequence of events	–	–	–
Abbreviations used in certifying the death	19.8	5.4	72.7
Absence of disease time interval	74.7	42.3	43.3
Additional errors on the certificate	5.3	5.1	3.7

Total column percentages may total > 100% as a single MCCOD may contain more than one error

^–^No data available

*Negative values indicate lower quality after training

^†^Category may contain minor errors

### Online system and basic training strategy

For the evaluation of the intervention in Peru, a total of 2100 MCCODs were assessed: 300 from baseline, 900 after the online intervention only, and 900 after the online and training intervention. At baseline, 100% of the certificates assessed were incorrectly completed, decreasing to 70% after the online intervention and 57% after the online and training intervention (Table 4). There was a decrease in all major errors after the implementation of the online intervention and a greater decrease in all major errors following the online and training intervention. The greatest improvement in minor errors occurred after the online intervention, with only a small additional improvement evident in some errors after the online and training intervention.

**Table 4:** Quality assessment of medical certificates of cause of death pre- and post-training in Peru (online system and basic training strategy)

Assessment	MCCODs with errors (%)				
	Baseline	After online intervention	After online and training intervention	Percentage improvement after online intervention*	Percentage improvement after online and training intervention*
Incorrectly completed certificates (at least one error)	100.0	69.9	56.7	30.1	43.3
Certificates with one error	16.3	18.2	18.6	− 11.7	− 14.1
Certificates with two or more errors	83.7	51.7	38.1	38.2	54.5
Certificates with at least one major error^†^	69.0	53.4	44.0	22.6	36.2
Certificates with minor errors only	31.0	16.4	12.7	47.1	59.0
Certificates with major errors					
Multiples causes per line	2.0	1.3	0.6	35.0	70.0
Incorrect sequence of events leading to death	40.3	25.9	17.9	35.7	55.6
Illegible handwriting	–	–	–	–	–
Ill-defined condition entered as an underlying cause of death	52.0	45.4	38.9	12.7	25.2
Additional information on external causes not available	5.0	4.5	2.1	10.0	58.0
Additional information on neoplasms not available	15.0	8.1	6.3	46.0	58.0
Certificates with minor errors					
Presence of blank lines within the sequence of events	11.3	0.2	0.3	98.2	97.3
Abbreviations used in certifying the death	11.7	4.6	4.1	60.7	65.0
Absence of disease time interval	96.0	47.1	30.0	50.9	68.8
Additional errors on the certificate	12.7	13.9	12.6	−9.4	0.8

Total column percentages may total > 100% as a single MCCOD may contain more than one error

^–^No data available

*Negative values indicate lower quality after training

^†^Category may contain minor errors

## Discussion

This study assessed the quality of medical certification in five countries, pre- and post-implementation of specific training strategies. In all countries, training physicians in medical certification improved the diagnostic accuracy of certification; the proportion of incorrectly completed MCCODs ranged from 73 to 100% prior to training, decreasing to 44–75% post-training. Despite these improvements, it is notable that 23–45% of certificates still contained major errors post-training. Whilst direct comparisons between countries are difficult to interpret due to significant economic, cultural, and health system structure variations, the experiences suggest that certain aspects of country strategies are likely to have had a greater impact on diagnostic outcomes than others.

### Training of trainers strategy

The main training intervention involved training of physicians as master trainers through a TOT approach; however, variations existed across countries in this training strategy. In the Philippines, the evaluation of pre- and post-assessments from the hospitals showed the greatest improvement in the accuracy of medical certification of COD of the three TOT countries, with a 40% increase in error-free MCCODs 6 months after training. However, this may still be less than expected for the completion of a relatively straightforward form, and there are several reasons why this may be the case. Firstly, the high turnover of physicians and medical record staff affected the retention of MCCOD knowledge within the hospitals. Secondly, regarding training courses, even the trained master trainers had difficulty getting time off from their regular clinical work to deliver these programmes. A lack of refresher trainings and trainings for new staff both likely affected the retainment of MCCOD knowledge within target hospitals. Other key factors might have been the lack of regional or central government monitoring of the training and the lack of incentives to the participating hospitals.

In Sri Lanka, there was only a 28% increase in error-free MCCODs when assessed 6 months to 2 years post-training. Again, there are several possible explanations. Five master trainers were trained from each hospital to train the intern medical officers in their hospitals. However, although there was interest, physicians found it hard to stay for the duration of the training as they were often summoned for urgent clinical duties. Furthermore, the master trainers were themselves junior physicians and therefore not in a position to train their seniors and consultants.

In Myanmar, there was a 33% increase in error-free MCCODs, and there are several reasons why this may not have been higher. Some master trainers did not spend adequate time for multiplier training sessions as required by the standard manual; in particular, they did not use case scenarios to practise MCCOD skills. Refresher trainings could not be conducted to further improve the knowledge and skills of doctors. In addition, the most appropriate physicians were often not selected as master trainers. Our experience training physicians in hospitals suggests that relatively junior staff often attend such trainings, but that these individuals may not be the most suitable to ensure good coverage of training in the hospital—or acceptance by senior staff—especially without significant prior sensitisation from other senior medical or government staff. There was also a significant turnover of trained doctors, with many, including master trainers, transferred to non-target hospitals. Final factors potentially impacting improvements in MCCOD quality in Myanmar include varying procedures for notification and sharing of hospital COD between hospitals and even between wards within the same hospital and that adequate systems to review the quality of MCCOD data from hospitals and provide feedback to doctors could not be implemented during the intervention.

### Direct training strategy

In PNG, training was provided by UoM D4H staff directly to certifiers in specific major hospitals. Initially, master trainers were trained, but further roll-out trainings were not conducted. This may in part have been due to this being a new intervention still lacking overt high-level support from national and provincial health administrations. The direct training strategy started as the opportunistic training of physicians when they gathered, for example, at symposia, but this was deemed an unsustainable strategy to train high numbers at any facility or change the culture regarding medical certification in the health system hierarchy. Monitoring and evaluation of ad hoc trainings in this manner was also not possible, an important factor in the decision to alter the strategy to focus on directly delivering training at the main hospitals.

There are clear differences between the training provided by local master trainers and by external experts. Whilst the quality of training by well-trained country master trainers should be similar to external (e.g. UoM D4H) trainers, country-led trainers benefit from the ability to use local examples and acceptance and engagement with trainees, including overcoming language barriers. However, the motivation of local master trainers and quality of training have the potential to vary more than when providing training directly to hospital staff. Well-organised supervision is required to ensure functional training programmes, but this varies considerably depending on senior-level buy-in to the MCCOD agenda. To facilitate sustainability through local monitoring and ownership of the MCCOD agenda, UoM D4H encouraged the formation of provincial committees based at each provincial hospital that would review a selection of MCCODs on a monthly basis. The institutionalisation of these committees to regularly monitor quality and provide feedback to individual certifiers is part of the ongoing intervention in PNG and is likely to be a key factor in maintaining and enhancing improvements produced by MCCOD training. Similarly, as part of the initial TOT strategy, existing master trainers were tasked to undertake regular monitoring of MCCOD quality at the hospital level, with feedback to certifiers.

### Online system and basic training strategy

The online SINADEF system introduced in Peru enabled an improvement in certification quality through several mechanisms: (1) eliminating the effect of illegible handwriting, (2) reducing the use of ill-defined conditions by having warning ‘popups’, and (3) not allowing for blank spaces to be left between the lines of events. SINADEF also greatly improved the timeliness of data, as online certificates became available immediately to the Ministry of Health [19]. Further improvements in the quality of certification were evident, particularly in reducing major errors, after the training of physicians in correct medical certification in addition to the use of the online system.

The experience from Peru highlights that both the introduction of electronic systems and specific training for physicians can lead to improvements in the quality of certification. The training courses supported by D4H in Peru, although brief, provided an understanding of the public health importance of correct completion of MCCODs, in addition to training on the procedure itself. From our experience, to improve best practice, we believe it is at least as important to change attitudes towards medical certification in hospitals as it is to train staff in the procedure and that even 1- to 2-h training sessions, such as those implemented in Peru, can still lead to improvements in the quality of certification. It is likely that further improvement would be evident with a half- to 1-day training course.

### Institutionalisation for improved MCCOD practice

The institutionalisation of medical certification practices and the trainings at the national level and within the hospitals are essential pre-requisites for the sustainability of an MCCOD programme within a country. The interventions described here were implemented in collaboration with national governments, with the majority of funding from D4H, but with ongoing discussions regarding full country ownership of the programmes and the best methods to ensure sustainability. The institutionalisation of MCCOD training at the national level, for example, through introduction into the medical curriculum, is an important route to sustainability and institutionalisation. This was achieved in three of the five countries included in this evaluation. In Sri Lanka, five physicians (whose only mandate is training) from the National Institute of Health Sciences, the premier training institute under the Ministry of Health, were trained as trainers in MCCOD. In Myanmar, professors and lecturers from five medical universities and two public health universities discussed how to teach MCCOD to medical students in their preventive and social medicine and forensic medicine curricula and pre-service trainings, and in PNG, MCCOD training was introduced into the curriculum in one of two medical schools in 2018, with a commitment to introduce it into the second in 2019.

Institutionalising mortality committees within hospitals is an effective way to achieve oversight of certification and training. Despite this being a planned component for all sites from the beginning of the intervention, it was not possible to establish such committees, due in part to inadequate advocacy with hospital administrators to build support for the MCCOD intervention. In a context of high physician turnover, refresher trainings and proper monitoring and evaluation of the MCCOD programmes are required within hospitals to ensure effectiveness and sustainability of the intervention and to generate a culture supportive of improving MCCOD data. Transfer of doctors and certification by doctors who only rarely complete death certificates are expected to become less of an issue with increased institutionalisation, especially the inclusion of MCCOD in medical training.

From our experience working in countries, the first major step towards improvement in certification is achieving buy-in at senior levels of government and in hospitals through advocacy meetings that can impart an understanding of the policy and programme importance of COD data and how their utility can be improved through better quality routine certification. The use of local data, from pilot studies and early monitoring of interventions, has been an effective tool to guide these discussions and promote MCCOD trainings. Government buy-in is essential to establish a sustainable framework and expectation to support medical certification, and buy-in from senior physicians is required to promote a cultural change in hospitals regarding attitudes towards medical certification. For both government officials and senior physicians, reviewing the quality of COD data produced at both a national and hospital level has proven helpful in generating the crucial support for the changes required to improve certification practices.

We note that the coding rules applied during high-quality manual coding and automated coding may address certification errors to some extent, but we strongly believe that the accuracy of cause of death statistics depends much more on improving the diagnostic acumen and skills of doctors who certify the cause of death by completing the MCCOD. Further, not all certification errors can be addressed at the coding stage, for example, even the positioning of disease conditions on a single line in part 1 of the MCCOD can affect the underlying COD selection. Our experience in the Philippines is that, even with automated coding, many problems caused by poor certification remain. Whilst improved coding and automation—as well as the introduction of smart electronic death certification systems that allow immediate feedback and direct interaction between coders and medical doctors—may produce additional benefits in the longer term, sustainable improvements in the quality of medical certification, such as training programmes and introduction into medical curricula, are likely to have the greatest impact on improving the quality of COD data over both the short- and long term and hence their policy utility.

There are several limitations to the evaluations we have reported here, mostly related to the fact the activities were part of country implementation projects as opposed to research. The data presented here compare the quality of certification at baseline to quality post-training; for pragmatic reasons, a ‘before-and-after’ design was used rather than a randomised approach. Whilst this design may limit the certainty that factors other than the training intervention influenced the findings, by far, the most likely reason for the improvements observed in all countries is the provision of training. Indeed, for Peru, training on the online system only, without training in medical certification of COD, may serve as a control, with improvements in quality recorded even after brief training on medical certification.

It is likely that at all sites, some proportion of the MCCODs used as part of the post-training assessment may have been from physicians who had not received training, leading to an underestimate of the impact of training on the quality of certification. In addition, the time period covering the post-training certificates may have affected the assessment, as the impact of training may vary over time depending on the supportive frameworks in place (such as refresher trainings, monitoring, and feedback). As there were several trainings carried out in each setting, ranging from approximately 6 months to 2 years, the impact of variable timing of post-training assessment of MCCOD accuracy might have been considerable. Finally, this study did not include an assessment of the intervention on mortality distribution by underlying cause due to the relatively low number of certificates available. Moreover, the objective of this study was to test the effectiveness of the intervention on reducing common errors in medical certification, which is best measured directly by assessing completed death certificates.

## Conclusion

Whilst the strategies implemented by D4H all produced an improvement in the quality of medical certification of COD, there is clearly scope for improvement beyond that reported here. Changing attitudes in hospital practice may be difficult to achieve rapidly, but the evidence from the country interventions discussed here indicate that a variety of strategies can lead to improved medical certification, albeit less than expected or desired. Further efforts to rapidly improve the quality of medical certification of causes of death in countries should build on the lessons learned in this exercise, particularly around the need for careful and effective engagement of the health sector and hospital administrators, and the establishment of effective, but not onerous, practices to monitor intervention impact and amend as necessary. Implementation of systems to improve medical certification will be influenced by unique country requirements and constraints, but lessons from the countries presented in this paper, particularly regarding the engagement of high-level stakeholders, may still be drawn on to facilitate progress towards improving the quality of medical certification elsewhere.

A TOT approach, with sufficient prior engagement of key stakeholders, is likely to be the most effective, affordable, and feasible strategy, especially as part of a country-wide roll-out of a medical certification of COD intervention. Our experience strongly suggests that prior engagement of decision-makers in government and hospital management is required to develop an understanding of the importance of good-quality medical certification, alter the culture regarding MCCOD in hospitals, and provide the time for staff training. The TOT approach requires careful planning so that appropriate trainers are selected and sufficient supervision and monitoring of the programme is possible. Hospital-level routine assessment of MCCOD quality, feedback to certifiers, and regular refresher training, including for new staff, will help institutionalise a culture supportive of MCCOD and maintain an improved quality of medical certification.

## Data Availability

The data that support the findings of this study are available from the civil registration and vital statistics system of each country, but restrictions apply to the availability of these data, which were used under licence for the current study, and so are not publicly available.
